# Mapping Educational uncertainty stimuli to support health professions educators’ in developing learner uncertainty tolerance

**DOI:** 10.1007/s10459-024-10345-z

**Published:** 2024-06-13

**Authors:** Michelle D. Lazarus, Amany Gouda-Vossos, Angela Ziebell, Jaai Parasnis, Swati Mujumdar, Gabrielle Brand

**Affiliations:** 1 Centre for Human Anatomy Education, Department of Anatomy and Developmental Biology, Biomedicine Discovery Institute, Faculty of Medicine Nursing and Health Sciences, Monash University, Melbourne, VIC Australia; 2 Monash Centre for Scholarship in Health Education, Faculty of Medicine Nursing and Health Sciences, Monash University, Melbourne, VIC Australia; 3 School of Life and Environmental Science, Deakin University, 221 Burwood Hwy, Burwood, Vic 3125 Australia; 4 Department of Economics, Monash University, Wellington Rd, Clayton, VIC 3180 Australia; 5 School of Psychological Sciences, Monash University, Wellington Road, Clayton, Vic 3180 Australia; 6 School of Nursing and Midwifery, Monash University, 47-49 Moorooduc Highway, Frankston, VIC 3199 Australia

**Keywords:** Uncertainty tolerance, Qualitative methods, Curriculum, Health professions education, Pedagogy

## Abstract

Uncertainty is a feature of healthcare practice. In recognition of this, multiple health profession governing bodies identify uncertainty tolerance as a healthcare graduate attribute and evaluate uncertainty tolerance within new graduate cohorts. While it is clear that uncertainty tolerance development for healthcare learners is valued, gaps remain for practically addressing this within healthcare curricula. Guiding frameworks for practical approaches supporting uncertainty tolerance development in healthcare learners remains sparse, particularly outside of medicine and in certain geographical locations. As uncertainty tolerance is increasingly recognised as being, at least in part, state-based (e.g. contextually changeable)– a broader understanding of teaching practices supporting uncertainty tolerance development in diverse health professions is warranted. This study explored educators’ teaching practices for purposefully stimulating learners’ uncertainty tolerance. Semi-structured interviews investigated how academics at a single institution, from diverse fields and health professions, stimulate uncertainty across multiple learning contexts. Framework analysis identified three themes for stimulating uncertainty: *Purposeful questioning*, *Forecasting uncertainty*, and *Placing learners in unfamiliar environments*, with characterisation of these themes (and related subthemes) also described. Many of the identified themes align with aspects of existing learning theories suggesting that curricular frameworks supporting learner uncertainty tolerance development may be informed by theories beyond the boundaries of health professions education research.

**Supplementary Information:**

The online version contains supplementary material available at 10.1007/s10459-024-10345-z.

## Introduction

### Health professions education: the role of uncertainty tolerance

As uncertainty remains a feature of healthcare practice, health professions and educational institutions globally seek to prepare matriculating students for a dynamic and changeable workforce. Defined as “*a subjective perception of not knowing what to think or what to do*” (Sommers & Launer, 2014, p. 3), it is widely accepted that uncertainty is “*normal, understandable, and to be expected in professional practice*.” (Coles, 2013, p. 53; Moffett et al., 2021). In recognition of this, many health professional bodies and institutions are developing or calling for core competencies which either directly or indirectly relate to healthcare graduate’s capacity to effectively manage uncertainty, often referred to as uncertainty tolerance (AAMC, 2020, 2021a; APA, 2023; Ingvarsson et al., 2019). Reinforcing this notion, a 2012 systematic review exploring the needs of newly graduated health professionals found a wide call for new graduates to effectively manage uncertainty (Gibson & Molloy, 2012).

*Uncertainty tolerance* extends from the perception of uncertainty and is defined as how one responds to uncertain stimuli once perceived. Healthcare-related uncertainty stimuli, broadly, come from a variety of sources (Lazarus & Stephens, 2024) related to effective healthcare professional practice (McConaghy, 2006); the evolution of societal norms, values and priorities informing healthcare practice (Baker et al., 2021; Kapiriri & Razavi, 2022; Landwehr & Klinnert, 2014; Whyle & Olivier, 2020); and the sheer complexity of working within such dynamic healthcare systems (Begun & Thygeson, 2015; Cinaroglu, 2016; Leykum et al., 2014; McDaniel et al., 2013). Within the narrower context of patient care, stimuli of uncertainty can stem from the biomedical aspects of healthcare (e.g. patient presentations, diagnostic interpretations, prognosis (Bhise et al., 2018; Djulbegovic et al., 2011; Han et al., 2021; Strout et al., 2018) etc., the psychosocial aspects of healthcare (e.g. patient encounters and/or teamwork) (Lian et al., 2021; Miao et al., 2020), and from the challenges of supporting patients within the complex healthcare system.

While there are different conceptual models for how uncertainty tolerance manifests for individuals (Gerrity et al., 1990; Hillen et al., 2017; Lee et al., 2021; Scott et al., 2023), shared across these models is a source (i.e. stimulus) of uncertainty which is recognised by the individual, and factors (both intrinsic and extrinsic) which modulate or moderate an individual’s responses to the uncertainty stimuli. Together, features of the uncertainty stimuli and of the moderators impact an individual’s response to this uncertainty (e.g. their uncertainty tolerance) which manifests across their thoughts (cognition), feelings (emotions), and actions (behaviours). Within this modelling, one may respond more adaptively (e.g. more uncertainty tolerant) or maladaptively (e.g. less uncertainty tolerant). Adaptive responses to uncertainty include being able to acknowledge the uncertainty when present, act with the information available, and adjust as new information comes in (Lazarus & Stephens, 2024). At the other end of the uncertainty tolerance ‘continuum’, maladaptive responses to uncertainty are described in those that ignore the uncertainty or give up in the face of the uncertain stimulus. The uncertainty stimulus, therefore, is the initiation stage of an individuals’ uncertainty tolerance.

The way healthcare providers’ respond to these sources of uncertainty are varied (Lazarus, 2023). Thus far, the strongest evidence links doctors’ psychological wellbeing to their uncertainty tolerance; for example, doctors who are less effective in managing healthcare-related uncertainty are at greater risk for psychological distress and burnout (Cooke et al., 2013; Di Trani et al., 2021; Hancock & Mattick, 2020). While the causality between uncertainty tolerant and health care worker burnout is still be investigated, some studies also suggest a relationship between provider's uncertainty tolerance and their personal efficacy (Begin et al., 2022). Those who are less uncertainty tolerant may be more likely to blame themselves when uncertainty presents itself (Begin et al., 2022; Katsaros et al., 2014). There is also evidence that uncertainty tolerance impacts healthcare providers’ openness to treating underserved patients (Caulfield et al., 2014; Wayne et al., 2011), engagement with person-centred care (Geller et al., 1993), and their utilisation of healthcare resources (Lysdahl & Hofmann, 2009; Strout et al., 2018). Those with less adaptive responses to uncertainty appear less likely to engage with the healthcare needs of underserved communities (Caulfield et al., 2014; Wayne et al., 2011), more likely to enact paternalistic approaches to medical care (Geller et al., 1993), and more likely to over-order tests and investigations (Lysdahl & Hofmann, 2009; Strout et al., 2018).

What is clear from the literature is that developing healthcare learners’ uncertainty tolerance as they progress through their health professional education could have widespread positive impacts on the wider healthcare system. Considering the healthcare sector-wide workforce shortages (Harp, 2023; Kimo Takayesu et al., 2014; Peters, 2023), and the correlative link between uncertainty *in*tolerance and burnout, developing learning opportunities to support healthcare students’ capacity for effectively managing uncertainty now, and in the future, is imperative.

### Healthcare UT teaching practices: knowns and unknowns

While there are multiple calls for supporting healthcare learners’ in developing skills for effectively managing uncertainty (AAMC, 2015, 2021b; Cumming & Ross, 2020; Englander et al., 2013; GMC, 2018; Kim & Lee, 2018; Simpson et al., 2002; Toohey et al., 2002), gaps remain in existing research and recommendations for educators interested in supporting such endeavours (Lazarus et al., 2023b).

Current studies exploring health professions learners’ uncertainty tolerance primarily focus on a narrow subset of healthcare coursework, namely that of health humanities and arts courses (Lazarus et al., 2023b; Patel et al., 2022). Furthermore, the healthcare student context most often studied is that of medical students (Moffett et al., 2021; Papanagnou et al., 2021; Patel et al., 2022; Stephens et al., 2022a), and to a lesser extent nursing, at the expense of other health professions (e.g. physiotherapy, radiography, etc.). There are also research gaps in teaching practices which foster learners’ uncertainty tolerance outside of the geographical contexts of the UK and USA. With the increasing evidence that uncertainty tolerance has contextual components, (Han et al., 2021; Stephens et al., 2023b; Strout et al., 2018), it is important to fill these research gaps to better understand the different contextual factors influencing uncertainty tolerance development *across* health professions globally. Such research would serve to enhance the capacity for developing effective education aimed at building this health professions attribute.

With existing research limitations in mind, a scoping review to synthesize available literature about medical school interventions related to learner uncertainty tolerance development (Patel et al., 2022) did identify some educational uncertainty stimuli, such as: “problem-based learning (PBL); medical humanities; assessment; reflection; anatomy; clinical feedback; and tactical decision games” (Patel et al., 2022, p. 1169). However, the authors noted that “none of the identified studies explicitly stated the source of uncertainty utilised.” (Patel et al., 2022, p. 1169)– meaning that while generic learning activities were identified as stimulating learner uncertainty, the specifics about which part(s) of the learning activity is/are responsible for stimulating learner uncertainty remains unknown. Consider, for instance, PBL whereby educators are left to ponder whether the learner's uncertainty is stimulated by: the context and details of the PBL case?; the teamwork required to solve the case?; the environment the case is undertaken?; the facilitators impact on this environment?; Or a combination of each of these? Without this type of specificity about the educational uncertainty stimulus, it may be difficult for a health professions educator to adapt PBLs in a manner most effective for supporting learners’ uncertainty tolerance development.

Similarly, a 2021 scoping review (Moffett et al., 2021) focusing on a wider population of undergraduate health professions students (though predominately medicine) characterised types of uncertainty that health professions students encounter including: 1) “the practice of healthcare itself”; 2)“the educational process”; and 3) “the learner’s self”. The educational context within these learning experiences were also identified: (1) “when learners experienced differences between themselves and others”; (2) “unfamiliar situations, or issues lacking easily distinguishable solutions.”; (3) “at transitions”, in (4) “PBL [problem-based learning] teaching” and (5) “clinical environments.” This review goes on to identify specific learning contexts where uncertainty was stimulated including: “when engaging with topics such as professionalism, communication, ethics, clinical reasoning, evidence-based medicine, and interprofessional learning.”, suggesting that humanities-based topics and clinical reasoning coursework were rich locations for stimulating uncertainty. What is missing from these reviews, however, are ways for educators to adapt these findings to practical teaching approaches.

Some primary studies provide more guiding insight for educators interested in adopting uncertainty tolerance teaching practices. Stephens et al. (2020) identified that “grey cases”, where unknowns were purposefully built into PBL activities, stimulated uncertainty for medical students taking anatomy. Others have suggested strategies such as simulated scenarios or gamification (Drummond et al., 2016; Golden et al., 2018; Scott et al., 2020), peer or expert supported ‘dress rehearsals’ using in-depth case studies (i.e. Learning-by-Concordance (LbC) (Fernandez et al., 2016) and SNAPPS techniques (**S**ummarise history physical findings, **N**arrow the differential, **A**nalyse the differential, **P**robe preceptor about uncertainties, **P**lan management, and **S**elect case-related issues for self-study) (Wolpaw et al., 2009), modifying traditional single best answer assessments to allow for more formative style assessments (Sam et al., 2020), or utilising various reflective practices (Gowda et al., 2018; Nevalainen et al., 2010) can all serve as educational methods for stimulating *medical* students’ perceptions of uncertainty. What remains less well understood is to what extent these findings apply across educational and discipline specific contexts (e.g. outside of humanities courses and beyond medical education), and the applicability of these specific activities to a broader curricular framework (e.g. the impact of building in such activities longitudinally across the curriculum or degree pathway).

In addition to the teaching activities which stimulate learners’ uncertainty tolerance, the way in which educators deliver content (e.g. the method) also appears to play a role in medical students’ experiences of uncertainty. For instance, Lukšaitė et al. (2022) undertook a qualitative ethnographic study wherein they observed medical educators as they delivered foundational science teaching. This study identified that medical science educators engage a teaching strategy they referred to as the ‘medic filter’, where educators filter out uncertainty leaving only small portion visible to student. The motivation for this approach by medical educators often stemmed from structural factors such as time constraints, breadth of core/required knowledge within course, and gravity/high stakes nature of profession. This “simplification of complex phenomena through the ‘medic filter’”, as the authors phrase it, (Lukšaitė et al., 2022, p. 375), generates learners who view scientific knowledge as immutable facts as opposed to contextual and changeable. This study, in the context of health professions education, suggests that it isn’t just what (e.g. the teaching activity) stimulates uncertainty, but also how (e.g. teaching delivery method) uncertainty is introduced which remains germane for guiding health professions educators in fostering student's uncertainty tolerance development. For example, while the aforementioned review studies suggest that PBL may be a rich environment for stimulating uncertainty (Moffett et al., 2021; Patel et al., 2022), the Lukšaitė et al. (2022) study appears to suggest that if a medic filter is applied in facilitation of the PBL, the uncertainty stimulus may be cancelled out or the perception of uncertainty could be reduced, as opposed to such PBLs providing an opportunity to stimulate uncertainty for learners. Similar findings were identified in (Stephens et al., 2020, 2022b), where the educational approach of lecturing (e.g. didactic teaching) was identified to reduce perceptions of uncertainty in preclinical medical students. Herein, lectures resulted in students perceiving medical knowledge as known and knowable, limiting awareness of the uncertainties intrinsic in the body of medical knowledge.

Importantly, it appears that learners’ uncertainty tolerance can be developed through education. Stephens et al. (2020) found that learners cognitive responses progressed from ‘a belief in absolutes’ towards one that accepted uncertainty tolerance as a ‘professional competency’ following experiences in anatomy education (pg. 68), though emotional responses to the uncertainty stimuli persisted negatively (Stephens et al., 2020). In a follow-up study, Stephens et al. found similar results during medical students’ clinical years with cognitive and behavioural responses to uncertainty improving during rotations, and emotional responses remaining largely negative (Stephens et al., 2023a). From these collective studies, the authors suggest that emotional responses to uncertainty may not be the defining characteristics of ‘uncertainty tolerance’ because learners were able to think and act adaptively to uncertainty stimuli in spite of negative emotional responses.

Thus, studies so far suggest that education can be a valuable and impactful moderator of learners’ uncertainty tolerance, and provide some guidance about the types of teaching and learning activities which influence this. Most of the current literature focuses on the impact on learners' experiences and less on educators.Clearer instructive guidelines for educators are needed, particularly given the emotional impact on learners and the evidence that education influences learner uncertainty tolerance development. Given that this learning process begins with uncertainty stimuli, this study focused on exploring how, and to what extent, uncertainty stimuli can be introduced through teaching practices by exploring educators' teaching experiences.

Given the current gaps in the literature, and the value of uncertainty tolerance development for health professions students, this study sought to address the question: *What existing teaching practices do University educators draw upon for stimulating learners’ perceptions of uncertainty?*.

## Methods

### Human ethics

This study was approved by Monash University Human Research Ethics Committee (22,240).

### Site and participants selection

The data was collected from a single Higher Education institution (Monash University) that employs over 17,000 academic and professional staff, with nine global campuses. Data was collected from ‘practitioner-educators’ defined as either having worked in a given sector prior to their educator role or one who maintains this professional sector role while concurrently working in higher education (e.g. healthcare practitioner).

To explore best practices for preparing health professions students for managing uncertainty, participants were deliberately selected across a diverse discipline range to explore different uncertainty tolerance (UT) teaching approaches, methods, and activities for stimulating students' uncertainty tolerance. Overall, 36 participants volunteered to be interviewed from 6 faculties, which included Arts, Business and Economics, Education, Medicine, Nursing and Health Sciences (MNHS), Pharmacy and Pharmaceutical Sciences, and Science. Most participants were from the Medicine, Nursing and Health Sciences (MNHS) faculty, and included educators in a wide variety of healthcare fields including pharmacy, radiotherapy, nursing, midwifery, occupational therapy, nutrition, dietetics, physiotherapy, psychology, public health, occupational epidemiology, medicine, and biomedical research.

### Data collection

Semi-structured interviews (Online Resource 1) were conducted by authors (MDL, GB, JP, ED, SM and AZ) and designed to uncover the various methods educators use to stimulate uncertainty through their teaching practices. Interview questions were designed to explore the underpinnings of various teaching methods and how participants approach preparing students for workplace sector uncertainties. Interview questions were tested on a small sample of academics (*n* = 6) to ensure that questions were framed effectively, but no changes were required. Audio-only data was collected via Zoom (Zoom Video Communications, Inc., San Jose, CA) and exported for transcription to Otter (Otter.ai, Los Altos, CA), and cross checked manually by the second author (AGV) for any anomalies. Transcribed data was imported to NVivo (QSR International, Melbourne, AU) for full framework analysis.

All participant demographics were obtained in a survey completed prior to partaking an interview (Table 1– see below).

**Table 1:** Participant demographics

Interview number	Discipline/Faculty	Acronym	Gender	Role/Level	Length of Time (Mins)	Words
1–6	Business and Economics	*BussEco*	3 F 3 M	B– C	307	6444
7–9	Education faculties	*EdFac*	1 F 1 M 1NB	B - C	177	3057
10–31	Medicine, Nursing, and Health Sciences	*MNHS*	16 F 6 M	A– D	1193	21,808
32–36	Sciences	*Sci*	2 F 3 M	A - D	331	3762

NB: Participants within ‘Engineering’ and ‘Arts/Science (Sustainability)’ (i.e., two participants) were merged within the ‘Science’ discipline. Additionally, within FMNHS, the following specialities were reported; Nursing/Nursing and Midwifery *n* = 5, Pharmacy *n* = 4, Radiotherapy *n* = 3, Nutrition and Dietetics *n* = 2, Physiotherapy *n* = 2, Medicine *n* = 1, Occupational Therapy *n* = 1, Psychology *n* = 1, Public Health *n* = 3. In Australia & New Zealand, academic roles have letters associated with them so that Level A– Associate Lecturer; Level B– Lecturer; Level C– Senior Lecturer; Level D– Associate Professor; Level E– Professor

### Data analysis

The interviews (Table 1) lasted an average length of time of 55 min, with 34,097 recorded words analysed, in line with similar qualitative studies (Lazarus et al., 2023b; Stephens et al., 2022a). The data was assessed via abductive qualitative analysis (Lingard, 2015), where coding oscillated between deductive and inductive coding. The coding was initiated inductively. However, this study was part of a larger body of work exploring the role of education in developing learners’ uncertainty tolerance, and thus there were existing related codebooks (Lazarus et al., 2023a, b; Stephens et al., 2020) that were used to support the deductive coding. The theoretical lens for the analysis relied on the UT conceptual modelling (Hillen et al., 2017). For example, when codes extended beyond the existing coding framework and UT theory, inductive coding was employed. This abductive coding approach was undertaken using five-step Framework analysis, which included 1) familiarisation; (2) thematic framework identification; (3) indexing; (4) charting; and (5) mapping and interpretation (Gale et al., 2013; Lewis et al., 2003; Lingard, 2015; Smith & Firth, 2011). To start, the first phase (familiarisation) took place amongst authors (AGV and MDL), throughout the interview process, and later in the transcription cross-checking process. The next phase (2) included broad discussions relating the data to the existing UT theoretical model (Hillen et al., 2017) and codebooks (Lazarus et al., 2023a, b; Stephens et al., 2020) to determine themes within alignment, and/or extensions of these existing frameworks. From this, codes were identified and regularly discussed between A.G.V., G.B., and M.D.L. until a final codebook, inclusive of definition and quotes, was reached (Phase 3). The data was managed using NVivo 12 (QSR International, Melbourne, Australia), and was used to code all the data and facilitate Phase 4. Once coding was completed, a Matrix analysis was conducted to explore thematic relationships, specifically divergence and/or convergence of themes across disciplines (Phase 5). Phase 5 continued with exploring the extent to which identified themes align with existing learning theories.

### Team reflexivity

Before data collection and analysis commenced, all authors participated in a team reflexive exercise to promote open and honest communication, reflect on world-views, and gain insight into the team’s collective epistemologies and methodological orientation. In accordance with Barry et al. (1999), the purposes of the exercise were to encourage team members to share experiences, discipline and research interest and to reflect on their personal strengths and weaknesses surrounding the research topic and methodological approach.

All members of the team were involved with teaching students, though the disciplines and type of learners was diverse. Team members’ backgrounds included: psychology, anatomy, nursing, business and economics, and science and the team taught a range of students (undergraduate, graduate, and post-graduate learner populations) aiding in the capacity to understand the diverse participant disciplines. The research team had experiential insight into higher education, as all team members were academics, as well as deep knowledge of the students and institution. The team’s experience with qualitative methodology spanned from novice to expert, with some eclectic methodological research experiences and worldviews ranging from positivistic and quantitative to interpretivism aiding in the process of data analysis and interpretation.

## Results

Despite diverse data collection, discipline differences were not identified and educators’ methods used for stimulating uncertainty were shared across three themes: (1) Purposeful questioning and/or challenging students’ pre-conceptions; (2) Forecasting uncertainty; (3) Placing learners in unfamiliar environments.

### Purposeful questioning and/or challenging students’ pre-conceptions

In this theme, educators described identifying methods for confronting and contesting students' long-held beliefs. Challenging pre-conceptions was discussed as either being educator facilitated, and actioned through student-to-student peer discussion, or as a purposeful direct action by the educator. Some of the way’s educators challenged learners’ pre-conceptions included sparking debates/discussions amongst student peers, posing challenging patient scenarios, or having students discuss contentious social topics. Educators also described challenging misconceptions by “pushing” learners to explore their own learning experiences through reflective prompts, as this member of the Science faculty describes in relation to preparing science teachers for their future careers:

And so I asked students, what do you think works in a classroom? What worked for you? What teachers do you remember? What did they do? Why do you remember that? So really, really putting their learning experience, like getting them to filter through their own learning experience? And ask them, how do you want to run your classroom? Why do you want to do it that way? What’s going to work for you? (I32 - Sci)

This demonstrates how educators engaged learners in a critical reflective space, challenging learners to draw on their own experiences and think beyond what “should” or is “supposed” to happen in the learning environment towards acknowledging the complexity of workplace experiences.

### Forecasting uncertainty

Many educators described forecasting uncertainty by highlighting where ambiguity, complexity or unknowns are likely to present in future career contexts, stimulating uncertainty by challenging learner views that their chosen career has more certainty than it does in reality as illustrated by this quote from a BUSSECO educator who discusses the realities of the career ‘in the moment’ versus what the career is portrayed about in hindsight:

I will draw upon some of the things that I use in my teaching…the first is the example that if governments put into place certain policies, say during a recession, then that will help the economy to come out of a recession…[but] the governments, when things are happening in real time, it is very hard to know, to understand that you are in recession, to understand the extent of that recession, because the data always comes *after* things have happened. So, then the point at which the government’s need to make a decision for fighting recessions, they don’t have enough data to establish even the basic structures of: Where we are at? How bad the shock is?; Which sectors are affected?; Are we really in recession? and so on…And you can learn from the past, but this is not exactly the past situation. So it is very hard to know a priori how something would outcome you will get out of any business or economic decision. (I2 - BussEco)

This strategy moderated students' uncertainty tolerance by generating a ‘sense of purpose’ for learners’ (Stephens et al., 2022a) as they recognised the value of UT. This theme had multiple subthemes: including destabilisation using future career contexts; decision-making in the face of uncertainty; changeability of uncertainty; and grey cases;

For instance, an educator describes destabilising learners by highlighting how their chosen future career includes contexts that are uncertain:

for example, bringing that linkage to structural engineering and saying, well, you want to become a structural engineer, but you have to understand the uncertainty around environmental predictions. So, I’m linking those aspects because most students who come through the civil engineering degree have a very…a very singular mindset, whether they either want to do project management or just structural engineering. (I36– Sci)

While the'grey' in this following example from Law, sees the educator describing how judges are tasked with decision-making despite multiple possible scenarios and outcomes (i.e., decision-making in the face of uncertainty), and thus introduce learners to the reality of uncertainty in their future decisions:

We always tell them that it depends on the facts of the case, but also how the judge or judges have interpreted the facts and interpreted say, legislation and or a previous case in, in order to then reach an outcome in whatever the problem is before them. So sometimes the students find that hard if we’ve got two or say, three court cases in our textbook, on say, the same issue or similar facts. And the judges decide, in different ways. There are students who can find that very hard.... (I5– BussEco)

Furthermore, educators describe workplace scenarios by introducing a problem, describing the changeability of the knowns and unknowns as the situation evolves. Illustrating this uncertainty stimulus subtheme, an educator describes introducing learners to uncertainty by forecasting the changeable nature of uncertainty in a career in sustainability:

But then, as they go forward, it becomes more about what can we do about this kind of stuff…And that’s where not just this kind of overarching uncertainty about what the heck sustainability is, [as] you move through that uncertainty you find your place, but then it’s like, who do we need at the table? Do we need engineers and what they can offer? Or do we need physical scientists and natural scientists and what they can offer? Do we need social scientists? And how do we work with each of their skill sets to advance change? And so I think that’s what students end up I guess struggling with or, or productively struggling with is the bringing different kinds of knowledge to bear on problems and how to do that effectively. (I35– Sci)

Another source of forecasting uncertainty was the use of grey cases (Stephens et al., 2020), particularly introducing “grey” areas with authentic raw data, or by relying on existing real-world workplace examples. In the following quote, an educator describes stimulating uncertainty in nursing education both through a grey case (challenging students to consider how they would respond), as well as by challenging their preconceptions of what “nursing” is:

…but when we start bringing in scenarios for students as learners around something like…somebody with challenging behaviours, and when you’re trying to help that person, and they might be trying to hit you, or they might spit at you. And they say, dreadful things to you. Very personal things. And, and so how to teach the learner to manage the situation and then to manage the [patient]…and that makes them feel very uncomfortable, because it’s really incongruent, I think, with their ideas of who a nurse is and what a nurse does and how patients are supposed to behave… (I16 - MNHS)

### Placing learners in unfamiliar environments

The final strategy shared and described by educators for stimulating uncertainty was placing learners in an unfamiliar environment and included fieldwork, work-based placements, internships, practicums and even travel abroad opportunities designed to harness the diversity and intrinsic unpredictability of these “uncontrolled” contexts. These unfamiliar environments provided learners an opportunity to experience and practice managing uncertainty in preparation for their future workplaces. Some placed this later in the learner’s degree pathways, scaffolding the extent to which uncertainty is experienced by the student, as described in this quote about Science:

So, at first year, the experiments are tailored where, yes, there are variation there, there is variation there. But, you know, this the sole, the main trend is clearly observable, whereas its second year, you know, you observe more variation. And then at third year, you’re actually observing biological events as they occur in nature. So, you get the full gamut of variation. I think it’s more about the degree of variation will increase. (I33 - Sci)

Other unfamiliar environments included the learner population, with interprofessional learning described as stimulating uncertainty, for both the learner and the educator:

With the interprofessional learning day, the nurses are placed with medical students who basically never learned together. This puts them into really confronting situations and they [the situations] change depending on the day you do them. I went and [facilitated], and it was confronting for me. And I’m like, Oh, I did not know at all, how would I respond in that situation? And knowing there’s no right and wrong, and it doesn’t matter, because no one’s going to tell anyone else because they’re not allowed to. It sort of allows you to explore your own level of uncertainty. (I17 - MNHS)

### Dominancy of themes across disciplines

**Fig. 1:**
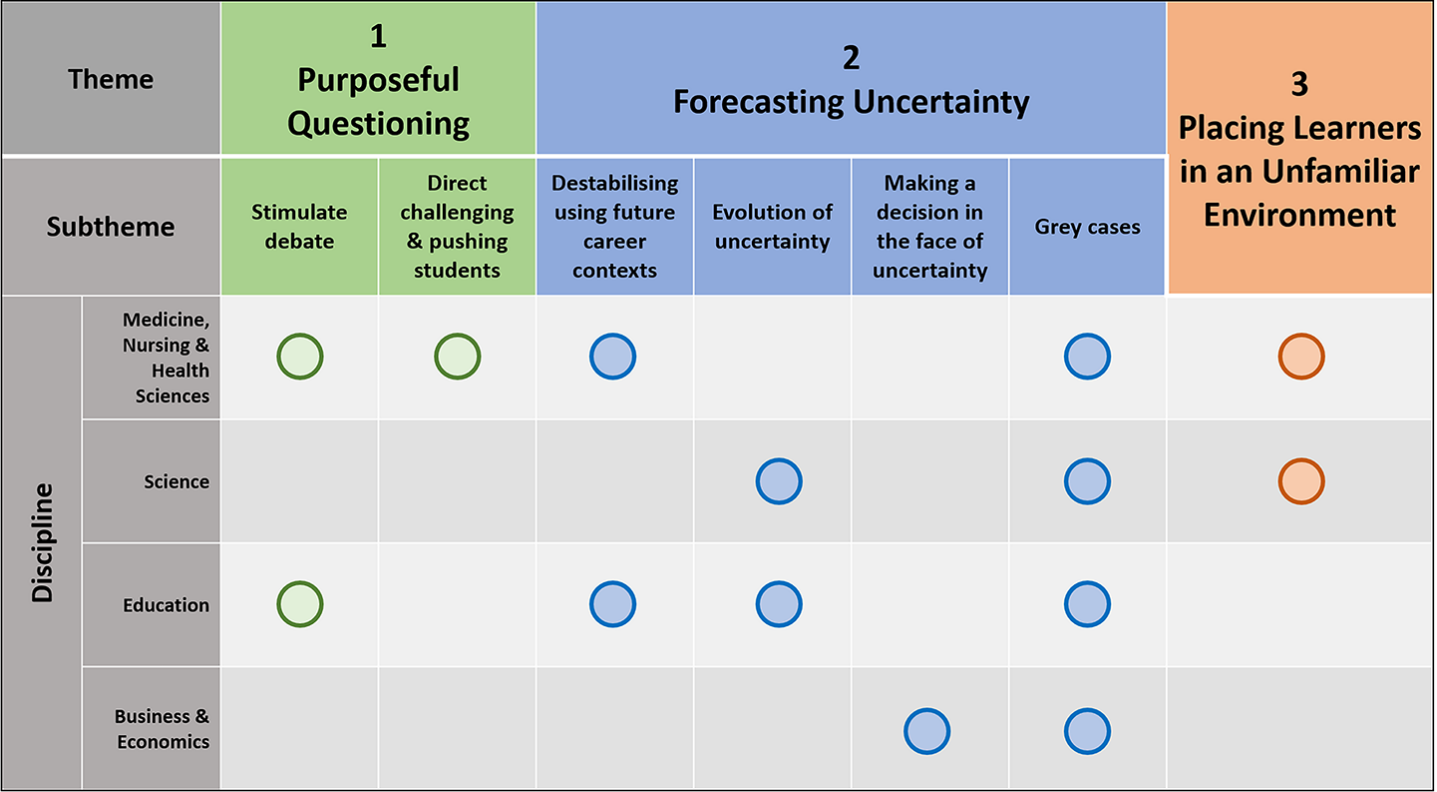
Matrix alignment exploring dominance of uncertainty stimuli themes and subthemes. Notably, all disciplines discussed dominantly engaging grey cases to stimulate learner uncertainty in their teaching practice

While all disciplines engaged each uncertainty stimulus theme, we explored the dominancy of these themes in each discipline using a matrix analysis. This cross-discipline comparison revealed, overall, synergy in methods used for stimulating uncertainty however there was variability in the dominancy of themes and subthemes across different disciplines (Fig. 1; Online Resource 2).

For example, education predominately reported stimulating uncertainty through *purposeful questioning and/or challenging students’ pre-conceptions* and *forecasting uncertainty* whereas business and economics predominately relied on *forecasting uncertainty*. Medicine, Nursing and Health Sciences (MNHS) had the most diverse engagement across the themes– with all three uncertainty-stimuli themes occurring across teaching practices, but participants described relying on *forecasting uncertainty* through *evolution of uncertainty* or *making a decision* in the face of uncertainty to a lesser extent. Science seemed to sit between MNHS and education with dominant themes expressed through *forecasting uncertainty* and *placing learners in unfamiliar environments*. Notably, the subtheme of *grey cases*, a known teaching practice which stimulates uncertainty (Stephens et al., 2020), was the dominant subtheme shared across all disciplines.

Because grey cases were dominant across all disciplines, we took a deeper look into the contexts used to generate these grey cases. *Real world examples* were used by all disciplines as the context for developing the grey cases to help *forecast uncertainty* in learners’ future careers:

I guess what it’s doing is…reinforcing the fact that it’s not a one-size-fits-all. It’s not black and white. This is the issue. This is the answer. It’s, it’s getting them to recognize the multiple factors that when someone walks in the door, there are multiple factors that you need to explore to understand why… (I19– MNHS)

*Grey cases* using *raw data* was used mostly in science, and Business and Economics, with educators stating that they purposefully provided incomplete data sets, or data with multiple variables that would stimulate the type of uncertainty they may face in their future health professional careers. As one educator stated:

You have to work with this information. So, we either give them the current year for some country, or even if we give them the past details, we always now give real data. And so, they can see all the problems one faces in real life about - there is incomplete data, there is… it comes in late, it has been revised, it’s not clean, things like that. So, it gives them real sense, instead of giving them some hypothetical clean series. (I2 - BussEco)

It should be noted that while this study took place during the rapid transition from face-to-face to online learning approaches during the COVID-19 pandemic despite the movement online, educators still described engaging uncertainty stimuli in remote teaching times.

For example, “*placing learners in an unfamiliar environment*”, was a minor theme in education, a discipline typically dependent on classroom-based placements and work integrated learning. Educators described how they converted a practical learning experience into an online learning experience during the COVID-19 pandemic where they stimulated discussion and debate amongst peers:

So in the practical term, for example, last year with online teaching, got them to share a video screen. And then the task would be to individually write down your first interpretations of that. So they do that individual task. And then there might be a breakout room is three or four in a breakout room. Okay, share your interpretation with the others. Right, fine. People, you had a similar interpretation with - people you’ve had a different one. You can sort of unpack that a bit. And then I might have like a shared document, like a Google Doc, and each group sort of puts up some of their thoughts. And then depending on time, we might actually come back or pick out each group, maybe two or three key things I want to share with the group about that. So yeah, just generally, it’ll be for drawing my own experience more I think a key one that they really need to be aware of as teachers. (I7– Edu)

Thus, while the emergency remote teaching appeared to impact *which* uncertainty-stimuli theme was engaged (in this example moving from placing learners in an unfamiliar environment to challenging preconceptions), it doesn’t appear to have affected whether uncertainty stimuli were introduced in the higher education context.

## Discussion

In order to explore how University educators stimulate learners’ uncertainty tolerance development we drew on data from across diverse disciplines to inform practical recommendations for teaching this core health professions graduate competency. This research expands our understanding of fostering uncertainty tolerance in health professional learners in several key ways. This work provides further characterisation and specificity for teaching practices that effectively stimulate uncertainty, leading to practical recommendations and by filling some critical research gaps, including discipline and geographical context.

### Mapping of educational stimuli of uncertainty

While prior work exploring uncertainty tolerance teaching practices focus heavily on arts and humanities courses in the medical student context (Bentwich & Gilbey, 2017; Haidet et al., 2016; Mangione et al., 2018; Moffett et al., 2021), our study illustrates that educators across multiple disciplines and diverse health professions *all* report engaging strategies for purposefully stimulating uncertainty. This finding suggests that educators across the gamut of healthcare curriculum, from every discipline and at every stage, can explore and engage in uncertainty tolerance teaching practices.

In health professions curriculum, the dominant forms of educational uncertainty stimuli identified included all three themes: *Purposeful questioning*; *Forecasting Uncertainty* and *Placing Learning in an Unfamiliar Environment*. The subthemes provide further insights into how these stimuli may be engaged practically in teaching.

Importantly, our findings provide more detail and direction for stimulating uncertainty by characterising *how* teaching approaches can be developed to support uncertainty tolerance teaching approaches. For instance, while others identified PBL as an educational context that can stimulate uncertainty, PBL may be most effective in stimulating uncertainty when the problem includes imperfect or raw/real-world workplace data or when the case challenges the learner to redefine their perceptions of the healthcare providers role, values and priorities. In this way, PBL alone may not stimulate uncertainty, without ingredients for effectively stimulating uncertainty which include how the PBL is designed and delivered.

While we found shared themes across how educators stimulate uncertainty (e.g. challenging pre-conceptions), the predominant way different educators used these stimuli varied, suggesting that discipline-specific knowledge guides the context of how educators stimulate uncertainty. For example, in education, stimulating debate and discussion amongst peers was the dominant forms of educational uncertainty stimuli, whereas MNHS reported direct challenges, including pushing students outside their comfort zone as their primary method for stimulating students' perceptions of uncertainty. This contrasted with business and economics who most often reported stimulating uncertainty through requiring decisions be made in the face of uncertainty. Despite this discipline-specific variation in theme dominance, all disciplines engaged all three major themes (and subthemes) for stimulating uncertainty, suggesting that healthcare educators can look outside of the health professions context for pedagogical inspiration in uncertainty tolerance teaching practices. This adds to evidence from our prior study that disciplines outside of healthcare may be valuable for supporting uncertainty tolerance teaching efforts, including in the humanities, arts, and social sciences (Lazarus et al., 2023b).

The advantage of exploring how these educators in different fields stimulate uncertainty is that we can draw on their experiences to inform our own healthcare teaching. For instance, we may want to expand our approaches to *forecasting uncertainty* by signposting the unpredictability of future health professions careers, instead of using approaches like the medic filter that supress uncertainty (Lukšaitė et al., 2022). Forecasting uncertainty could also be strategically included in *grey cases* predominantly used by healthcare educators. For instance, a patient case that highlights uncertainty around the edges of biomedical ‘facts’, as well as foreshadowing the role of uncertainty in learners’ future careers or including the *changeable nature of uncertainty* by altering the grey case so that once one aspect of the case is ‘solved’, another uncertainty is introduced. Such teaching practices would allow students to more readily grapple with the realities of *decision-making in the face of uncertainty*. By drawing on a wider educational landscape of teaching practices which introduce uncertainty stimuli more frequently in our teaching, health professions educators can better represent the diverse sources of real-world healthcare uncertainty learners will face in their future careers.

### Applying findings to health professions education

When educators purposefully introduce an uncertainty stimulus into health professions education, it allows students the opportunity to practice managing uncertainty. We found parallels with identified uncertainty stimuli themes and Mezirow’s (1994) first step of transformation in the transformational learning process. This first step in transformative learning theory is characterised as an event which is complex, unknown and/ or unpredictable (; Mezirow, 1994), and is often referred to as a ‘disorienting dilemma’. The similarities between uncertainty stimulus themes identified in this study and the 1st step of transformative learning extends to the subthemes identified. For example, in transformative learning theory, a disorienting dilemma begins when learners’ unexamined assumptions or frames of reference are challenged which triggers a self-examination and critical reflection of previously held beliefs (perspectives, mindsets) that are no longer functional (Mezirow, 1994), aligning with our subtheme of directly challenging and pushing students.

**Fig. 2:**
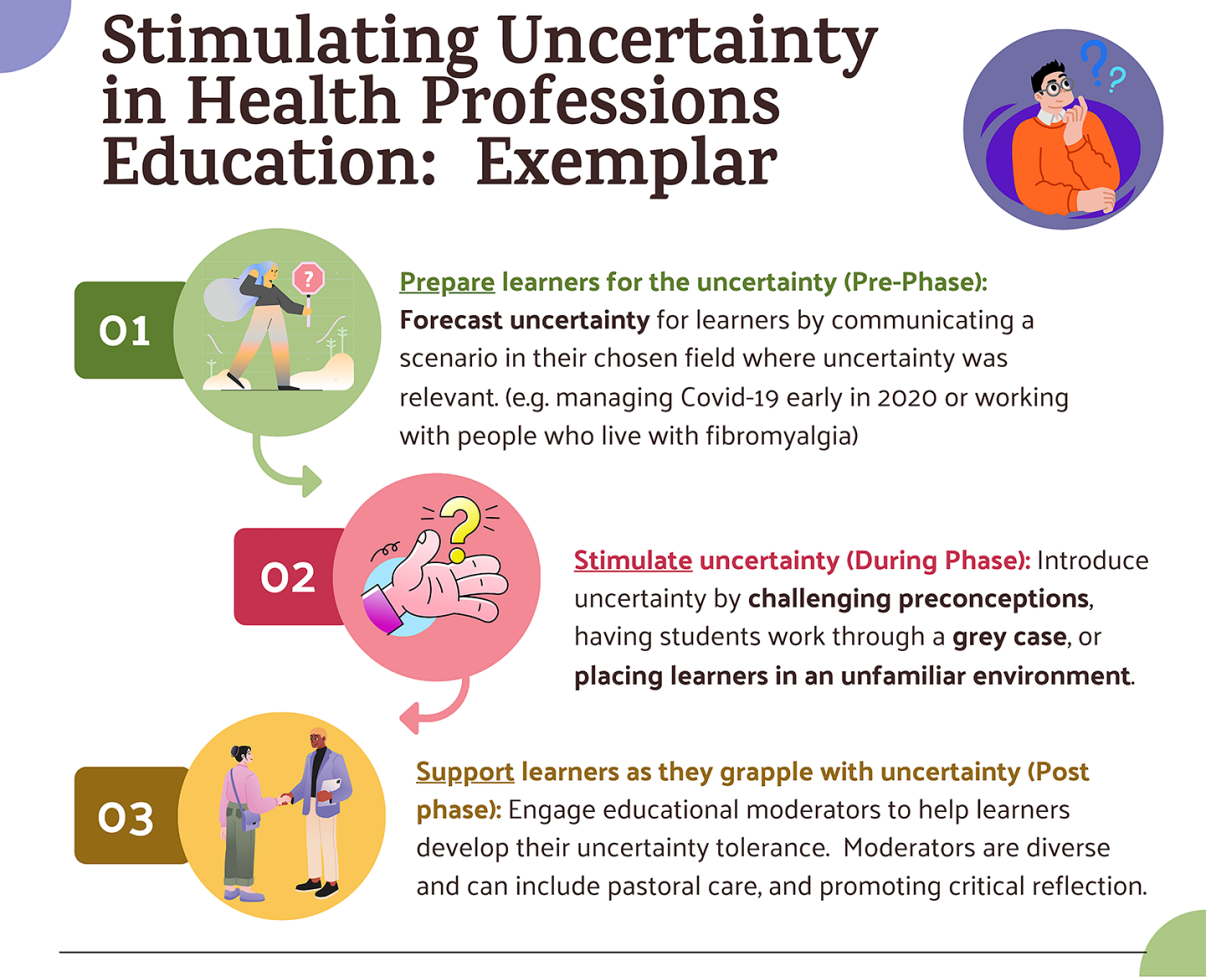
Stimulating uncertainty in Health Professions Education Applied to Transformative Learning Theory. Bolded terms are themes identified in this study

With this in mind, once health professions educators stimulate learners’ uncertainty, transformative learning theory suggests that it is vital for educators to support learners’ self-examination, exploration and forward planning. Transformative learning literature also suggests that disorienting dilemmas are not a singular event but rather a series of “pre”, “during” and “post” phases that includes a preparatory ‘crisis’ which sparks the self-exploration (Laros et al., 2017) phase. Given the similarities between the findings in this and other studies related to uncertainty tolerance development in learners and that of the transformational learning process, applying this three-phase transformative learning approach to health professions education could be of value (Fig. 2).

The first phase, the ‘scene setting’ or ‘pre-disorienting’ phase, appears to closely represent the theme *forecasting uncertainty*, identified in this study, by challenging healthcare learners early in their training to consider the tensions between their current view of healthcare to the actual reality. During this initial preparatory phase of managing uncertainty, learners may then move towards the disorienting dilemma as they begin to recognise the differences between their expectations and and reframe their reference points prior to transitioning to professional healthcare practice.

The second phase of transformative learning is the “during” phase, where educators can introduce further uncertainty stimuli through teaching activities and methods. This study highlights how educators can introduce uncertainty stimuli in this "during" phase in a manner well-suited to health professions learners, such as through challenging preconceptions, having learners work through grey cases, or through their experiences in clinical placements.

Transformative learning theory uses strong language, such as ‘disorienting’ and ‘crisis’ (Mezirow, 2000) to describe the “during” phase of learners’ transformation. This strong discomfort is similarly described in the uncertainty tolerance literature within medical student populations (Stephens et al., 2020, 2023a). As learners experience the uncertainty stimulus and move towards developing skills to effectively manage uncertainty (e.g. uncertainty tolerance), educators need to be aware that support for learners becomes critical to manage this psychologically challenging phase.

The final phase helps support learners as they grapple with uncertainty. Multiple teaching practices (e.g. moderators) are identified as important for managing learners’ discomfort after the uncertainty stimulus is perceived (Lazarus et al., 2023b; Stephens et al., 2020, 2022a). For instance, intellectual candour (Molloy & Bearman, 2019), where educators help learners adaptively respond to uncertainty by sharing their own related experiences with managing uncertainty, is shown to have a moderating impact on learners uncertainty tolerance development. Pastoral care, where educators work to create psychologically safe environments (Stephens et al., 2022b), has also been identified in helping learners manage uncertainty. One of the most consistent moderators supporting learners in managing educational uncertainty appears to be critical reflection which can occur in the form of a debrief in the clinic or a diary entry during preclinical course work (Lazarus et al., 2023b; Stephens et al., 2020, 2022a). Given the psychological distress that accompanies experiences of uncertainty stimuli, a well-considered learning environment for managing uncertainty is critical.

Further research could more deeply explore the relationships between uncertainty tolerance and transformative learning theory, focusing on uncertainty tolerance teaching practices which foster and hinder learners’ uncertainty tolerance development and further inform the development of effective uncertainty tolerance curricular practice across multiple healthcare disciplines.

### Strengths & limitations

This study’s strength includes the focused research question on uncertainty stimuli, purposive sampling of diverse educators, interview data depth and breadth, and alignment with existing theory (uncertainty tolerance conceptual modelling) that suggests adequate information power (Malterud et al., 2016). Furthermore, the authors engaged in reflexivity to help explore the perspectives from which the analysis was undertaken (see methods) and support the rigour expected of qualitative research (Barry et al., 1999; O’Brien et al., 2014). However, this study did not include evaluation of direct observations of teaching practices, and thus this study explored educator reflections of their own teaching practices which may not represent, fully, the actual experiences in the moment. This study was also conducted at a single institution, and thus may not represent a universal truth nor translate wholly to another context. However, the engagement of the above-mentioned theories, and the replication of some findings in other work, does suggest that aspects of this study may be applicable outside the study context (Firestone, 1993).

## Conclusions

This study extends our understanding of potential teaching practices which can be purposely integrated into healthcare education curricula to stimulate uncertainty beyond geographical boundaries and/or those described for medical students. The identified educational uncertainty stimuli seem to align with the first step in transformative learning theory, suggesting that drawing upon this theory for informing uncertainty tolerance curricular development and implementation may be useful. Indeed, such transformative learning approaches are explicitly highlighted as imperative for preparing health professionals for contemporary and future complex and uncertain workplaces (Frenk et al., 2010).

## Electronic supplementary material

Below is the link to the electronic supplementary material.

Supplementary Material 1

Supplementary Material 2
