# Supplementary material for: Mapping Educational uncertainty stimuli to support health professions educators’ in developing learner uncertainty tolerance
Source: Adv Health Sci Educ Theory Pract. 2024 Jun 13;30(1):259–80. doi: 10.1007/s10459-024-10345-z (PMC11925988; doi:10.1007/s10459-024-10345-z)
Supplement: Supplementary file 1 — Supplementary Material 1 [file 10459_2024_10345_MOESM1_ESM.docx]

# Online Resource 1

## Focus Group/Interview Facilitator Prompts

## Preamble

**1. Welcome**

- Hello! Thank you for making the time to meet today and for agreeing to this interview
- As I mentioned in the email, the purpose of this interview is to further discuss your experiences in higher education around teaching students to manage and prepare for uncertainty.

**2. Introduction**

- Introduce yourself, and ask the interviewee to introduce themselves.

**3. Anonymity and Right to Withdraw**

- The discussion will be audio and video recorded.
- After the discussion, the recording will be transcribed.
- All data will be deidentified prior to publication.
- I would also like to remind you that you have the right to withdraw at any time.
- If you do not wish to answer a question, you do not have to. If you find that any of the issues we cover are upsetting, as a Monash employee you can access the university employee Counselling Services.

**4. Ground rules**

- Before we begin, it is a good idea that we set some ground rules.
- Due to the potentially sensitive nature of our conversation it is important that you are careful not to disclose the names or identifying characteristics of other people involved in any situations you discuss.
- Everything that you say in this Zoom meeting will be kept anonymous and, while we will be reporting the findings in general, personal confidentiality will be maintained, with the exception of circumstances where it is likely to cause harm to you or to others. In this case, we will contact the appropriate authorities but we will inform you first.
- Due to the nature of Zoom, we ask that questions be answered through verbal communication, and not via chat.
- There are no right or wrong answers, everyone’s experiences and perspectives are valid.
- Finally, do you have any questions you would like to ask me about the focus group/interview or study in general before we start?

## ***START RECORDING NOW IF EVERYONE CONSENTS***

***[Interviewer Notes: Stimulus, Moderators and Responses may all come out at the same time, or through the same story. This is fine and expected.]***

## Interview Questions

### *Intro: For the purposes of this research, the definition of ToU/ToA we are working from is: the way an individual (or group) perceives and processes information about ambiguous situations or stimuli when confronted by an array of unfamiliar, complex, or incongruent clues.*

### Definition

1. **How do you define uncertainty within your discipline**?
   1. Do you agree with our definition? if so Why? If not? Why not?
2. **Could you provide an example (or two) of uncertainty that occurs within your field that you think learners need to prepare for**?

### Stimuli

1. **What do you think stimulates students to experience uncertainty within your teaching environment**? [goal: address stimuli of uncertainty in Higher Education]
   1. How do you introduce the concept of uncertainty into your classroom?
   2. Does this change over time? (throughout the semester or course?)
2. **Are there any active teaching approaches or practices that you purposefully engage to stimulate or create uncertainty in your classroom (ask them to describe these)?**
   1. Many in your faculty use [refer to table], do you use similar techniques? [**Interviewer note: These are prompts to help explore deeper**]
      1. Arts: Groupwork, portfolios, flipped classrooms
      2. BUSSECO: Groupwork and flipped classrooms
      3. Education equally use: Work-integrated learning, Simulation, Groupwork, portfolios, flipped classrooms [ask whether they prefer one over the other and why?]
      4. IT: Simulation and groupwork
      5. FMNHS: Groupwork, flipped classrooms, simulation (followed by WIL & Portfolios)
      6. Pharmacy: Groupwork
      7. Science: Groupwork
      8. OR STEM vs. HASS:
         1. STEMM: work integrated learning (WIL)
         2. HASS: Groupwork
   2. Have you considered others such as [refer to above]? If not, why not? If so, how would you use this in your own classroom?
   3. Could you describe what this looks like in the classroom?
   4. The way in which each faculty engages these general approaches (above) differs. What classroom strategies to you use to achieve this? **Could you describe what this looks like in the classroom?**
      1. Top: The majority of faculties top two were: Problems with ambiguous answers and focusing on process.
      2. Next tier: Unstructured tasks
      3. Third: Interdisciplinary learning, diverse student teams, changing direction, industry-based learning, future focused problems
      4. OR HASS vs STEMM:
         1. HASS: Interdisciplinary learning, unstructured tasks, future focused problems and creative artwork
         2. STEMM: Industry based learning more widely used.
3. **Do you think your unit/course/teaching helps students prepare for uncertainty within the field**? If so, how so? If not, why not (what are the barriers)?
4. **What are the barriers to teaching and fostering uncertainty tolerance in your unit, or across higher education**?
   1. University policy and procedures?
   2. Discipline focus?
   3. Classroom design?
   4. Individual factors (e.g. unaware or unprepared to teach this way?)

### Moderators

1. **Are there any practices or approaches you use in the classroom which help students manage/moderator/modulate their perceptions and/or responses to uncertainty? Please describe/give an example.**
   1. Some have described factors such as **pastoral care**, providing expectations around the discomfort, dress rehearsals of the real thing, intellectual candour – do any of these resonate with you? If so, can you describe how? If not, are there other factors that you think help (or hurt) uncertainty
2. **Can you describe an aspect of your curriculum which helps students manage uncertainty**? How did you design it? What does it look like?
3. **Have you observed any personal characteristics in EDUCATORS which help or hurt them manage uncertainties in the classroom?**
   1. Age?
   2. Prior experience with uncertainty pedagogy?
   3. Peers’ perceptions of the uncertainty curriculum?
   4. Discipline background?
4. **Have you observed any personal characteristics in STUDENTS which help or hurt them manage uncertainties?**
   1. Age (educator/student)?
   2. Prior experience with uncertainty?
   3. Peers’ perceptions of the uncertainty curriculum?
   4. Discipline background?
   5. Year level?

### Appraisal/Response

- **Thinking on the examples you provided earlier, what are some responses you have observed from students (and/or peers) to this uncertainty educational stimulus?**
  - **Emotionally?**
  - **Behaviourally (what do you see them doing?)**
  - **Cognitively?**
    - How do you manage these responses within the classroom?
    - Have you observed these responses in SETUs or in-class reflections? And what does it look like?
- **[Interviewer note: Prompt if positive responses to uncertainty aren’t raised]:** **Do you think uncertainty is ever something that students (or educators), enjoy or otherwise feel positively about? If so, can you describe this? What does this look like?**

### General Questions:

- **Could you describe the qualities of a student who is**:
  - Tolerant of uncertainty?
  - Intolerant of uncertainty?
  - And…what role do you think education has in affecting these two, if any?
  - What impact, if any, do you think reflection plays in students managing uncertainty?
- **What role do assessments play in UT? Are there aspects of assessments that hinder or foster UT within your discipline? If so - how? If not, what is a barrier**?
  - Formative vs. summative assessments
  - Tension between the rubric structure and the assessment [structure vs. vague]
- **Do you think UT pedagogy is essential in higher ed or something that should be left to the workplace? Why?/Why not?**
- **If another academic was trying to prepare their own students in development of ToA/ToU, what advice would/could you give them**?
  - What support is needed for academics in this space? What would this look like? (e.g. PD, examples, templates etc)
  - What role, if any, do you think pastoral care plays in managing students and educators through pedagogy related to UT?
- Has participation in this study impacted your understanding/awareness of uncertainty tolerance in higher education? If so, how?
- Do you have any examples of teaching or assessment that you didn’t attach to the survey that you would like to share (no pressure).
- If you think of anything after the interview, please send me an email.

### Closing:

- Do you have any follow up questions for me?
- If you have
- Are there any final comments, feedback or ideas that you would like to share?
- Thank you and closing.
