# Supplementary material for: Mapping Educational uncertainty stimuli to support health professions educators’ in developing learner uncertainty tolerance
Source: Adv Health Sci Educ Theory Pract. 2024 Jun 13;30(1):259–80. doi: 10.1007/s10459-024-10345-z (PMC11925988; doi:10.1007/s10459-024-10345-z)
Supplement: Supplementary file 2 — Supplementary Material 2 [file 10459_2024_10345_MOESM2_ESM.docx]

**Online Resource 2 – Dominant themes and subthemes of uncertainty stimuli with example quotations**

| **Education** | |
| --- | --- |
| **Dominant Theme/Subtheme** | **Example Quotes** |
| Purposeful questioning and/or challenging students’ pre-conceptions/**Stimulate Debate or discussion amongst peers** | I think by trying to reinforce that, we're trying to work in a safe environment where there isn't a right or wrong answer. There are better or poor answers…And just giving that example that, you know, sometimes they're the very answers that are productive, or they trigger others thinking about things I'd never thought of before. So, you know, in terms of brainstorming, or just going with your intuition or your gut feeling, doing that sort of front loading before the activity, but at the same time, telling them that they need to be able to justify their choices… |
| Forecasting Uncertainty/**Destabilising using future careers contexts** | …somebody making a racist statement, but some of them felt that that there wasn't enough stimulus around them for them to put themselves into that role. So the ambiguity was too great, but also they felt personally confronting rather than realising - Well, this is what happens in teaching these sorts of things. This is your place to practice your response because your response is what's going to be important here. |
| Forecasting Uncertainty/  **Subtheme: Evolution of uncertainty** | So if you're way I think about it, is that sort of like a roadmap that the students can have, and that can let them feel comfortable with lessons, which may be a slightly off where they want to go or go and slower, because they've got this kind of like, guide rails, but they've got sort of sense of, I know where I'm sort of going, I know that I'm going to be going a bit it looks like it's a bit slower but I know I can speed up a bit here. So there's uncertainty naturally in the learning. |
| Forecasting Uncertainty/**Grey Cases** | And then we went into scenarios where I guess, you know, trying to do the drama roleplay scaffolding didn't turn out to be enough for some of the students for some, it was fine. I went straight into role and they could deal with the scenario which might be an irate parent coming to your classroom, and swearing at you saying that you're trying to teach your kid how to have sex or something like that. And then how would you manage that? |
| **Business & Economics** | |
| **Dominant Theme/Subtheme** | **Example Quotes** |
| Forecasting Uncertainty/**Making a decision in the face of uncertainty** | …just helping them prepare for the workplace and the real world. And to give them the message to that, not everything's black and white. And there will be times say in legal issues, etc, say, with clients or customers in the workplace, that you might have to reach compromise . So part of this is that cases eventually, you know, there has to be an outcome, a dispute, etc, has to end at some point. And it might be that you have to compromise…. |
| Forecasting Uncertainty/**Grey Cases** | I also tried to bring the real world dealing, specifically. So that and specifically because it keeps it more relevant. And they and it tries to give them this is not irrelevant, because this is what is happening…I've turned around and said to them, the whole purpose of this is that in the end, if you can be aware of this to unpick these emotive statements that appear in the media, and not just go, “*Oh, yeah, they can't do that*”. But go, “*Well, why can't they do that?*” Then that's the purpose of really behind why we do it in a structured approach, rather than just doing it, you know, we can debate that it should be or it shouldn't be. The debate is, well, is this element missing? Is this element missing? Is this way the law allows the protection? That's where the debate is not the philosophical debate of does the law exists? Should trademarks really protect useless phrases? |
| **Medicine, Nursing and Health Sciences** | |
| **Dominant Theme/Subtheme** | **Example Quotes** |
| Purposeful questioning and/or challenging students’ pre-conceptions/**Stimulate Debate or discussion amongst peers** | we've allowed them to choose an ethical dilemma in healthcare, and then get getting to see it from different perspectives and how you would approach it from different perspectives using ethical theories and ethical principles. But our dilemma was students couldn't really understand/comprehend the ethical dilemma in healthcare. So now we've narrowed it down and we've provided them X amount of ethical dilemmas in healthcare, and we still get them to think about it from your perspective, it's a group assignment as well. And I think that allows them to have those conversations about being...that putting them in uncomfortable situations. |
| Purposeful questioning and/or challenging students’ pre-concept/**Directly challenging & pushing students** | they're talking about whether there's stem cells in the brain, and there were two papers that came out within a month, one said, Yes, there are stem cells that persist right through life, we can detect them all the way through and other papers that there's very little evidence for this. And so you can basically show the students that at the same time and say, What do you think? Yeah, I don't know. does that fit? |
| Forecasting Uncertainty/**Grey cases** | some of the students find that a bit difficult to get used to at the beginning. And then as the semester goes on this the case studies get more and more complex, and they get more and more realistic, and they deal with more and more issues at once. So by the end of the semester, usually, in the environmental health class I teach, at the end of the semester we end with a two week-two part case study on a failed built environment that is very, very far reaching, and I allow the students to go where they want to go with it. So if we have a class that's heavily weighted towards - happens to have a lot of regulators in it, they'll be that that that will be the flavour of the month, and that's fine. |
| Placing learners in unfamiliar environments/(n/a) | But we, a couple of years ago, we sent students to Sri Lanka to work in a psychiatric facility there. And last year, we send them to Bali, again, to work with in within one of these psychiatry facilities… Well, things that you take so much for granted in Australia…So it's kind of Yeah, so these things triggered a lot of, let's say, ambiguity that you have, if I work as a psychologist in one of these countries, you know. |
| **Pharmacy and Pharmaceutical sciences** | |
| **Dominant Theme/Subtheme** | **Example Quotes** |
| Forecasting Uncertainty/**Destabilising using future career contexts** | For example, our pandemic happened. And this also is a kind of source of uncertainty. So, but I guess more specific to pharmacy, it's these examples like medication, differences of patients that are difficult to deal with. And in this case, I guess our curriculum is to prepare them for these difficulties, or even to, even if we can, perhaps be better for every single uncertain situation, we can at least equip them accordingly. I have to tell them, okay, there, these are the references you can use. So if a patient comes to you with a job that you have no idea what it is, you actually know, you actually have the tools to find out. |
| Forecasting Uncertainty/**Directly challenging & pushing students** | Yeah, a lot of our clinician moderators will use sort of what if kind of scenarios within those discussions. So basically, as I described, like there's a there's a narrative is each student's allocated in a particular question. And we have changed some of the formats in that in the first one of these discussions they do or first set of them. All the students have to work through all the questions and develop answers to them, and then are asked to sort of elaborate on one answer each kind of thing within the discussion, which I think probably fits more with developing the perspective of there is some uncertainty in clinical practice, because they're, they know what they've said. And then their colleague who's answering that question may suggest something else. And in an ideal world, a nice debate and discussion in choosing that. Whereas for other two sets of therapeutic topics, we they only answer one question themselves, and so there's much more onus on them to engage with their colleagues after the fact, and sort of say, I would have done something different in that scenario, and things like that. So I think we're working towards it. I think it's great at the moment. |
| Forecasting Uncertainty/**Grey Cases** | …we use patient-centered or case-based teaching predominantly. And that's where we present students with us a narrative, not usually with a great amount of detail, probably just enough to make a basic decision. But then they're given a question that requires them to look beyond the obvious answer, and go to the primary literature to explore the different options if in different situations for that patient.…So that they need to sort of consider with the uncertainty of what if they were like this? What would we do? If they were like that? What would we do? How would I influence that? |
| **Science** | |
| **Dominant Theme/Subtheme** | **Example Quotes** |
| Forecasting Uncertainty/**Evolution of uncertainty** | So I guess, in biology, we're always exposed to natural variation.. So I think one way I prepare students for that is I introduce them to natural variation, and variation in biological phenomena, how that can be acceptable, and how we use statistics to account for that natural variation. So despite the fact that you might be seeing quite a bit of variation in your experimental results, statistics can help identify that there is a key trend in one particular direction. |
| Forecasting Uncertainty/**Grey cases** | I guess using real, real data. So it's not it's not specifically from samples that they've collected. But from samples that I've collected, for example, from, from previous research projects, …, that's always interesting, because then you also have the issue of other students, and the areas that they introduce, and what they've done previously. So, yeah, and so I guess sometimes I get them to compare their own data to a previous class data with the same issues that that previous class or that class is dealing in terms of dealing with in terms of uncertainty, but also the fact that it's student data as well, which, as we know, is challenging to deal with. |
| Placing learners in unfamiliar environments/(n/a) | I think they also they also come into this first-year unit and first year trip expecting to have these right answers for their field trip panned out. And so, they so what happens on this field trip is they have they have a field trip Handbook, and they rotate around stations that to collect our data to make observations and collect data, and then we mark on the fly as well. So they come up to us with a completed section and we disappear and we mark through and we mark them and i know some of them, they compare their workbooks and they compare what they've got and they and they sort of come back to us like well, this person has a different number to this. How can they both be right? Well, it's what it's what you measured. And if you look at the time of day, and you know where you are in the landscape and things like that. And then they sort of start to look at well, and then they start to sort of dig a little deeper. It's like, Well, why does that happen as well, we don't really know, at this point, because you've got all these other variables that we don't have the information for, |
